# Protocol of a dyadic, app-supported collaborative care intervention trial for informal caregivers of people living with dementia—the multi-center *living@home* study

**DOI:** 10.1186/s13063-026-09639-x

**Published:** 2026-03-18

**Authors:** Eva Gläser, Fabian Kleinke, Milena Armanowski, Maresa Buchholz, Andreas Fellgiebel, Marius Gerdes, Wolfgang Hoffmann, Bernhard Holle, Ingo Kilimann, Kerstin Köhler, Stefanie Köhler, Franziska Maier, Ingo Matthäus, Nils Pfeuffer, Anika Rädke, Dorota Sarwinska, Björn H. Schott, Frank Schwärzler, Enno Swart, Stefan Teipel, Neeltje van den Berg, Tim Wendisch, Jens Wiltfang, Alexandra Wuttke, Bernhard Michalowsky

**Affiliations:** 1 German Center for Neurodegenerative Diseases (DZNE), Site Rostock/Greifswald, Greifswald, Germany; 2 Institute for Community Medicine, University Medicine Greifswald, Greifswald, Germany; 3 AOK Niedersachsen, Hannover, Germany; 4 Department of Psychiatry, University Medical Center of Mainz, Mainz, Germany; 5 Karlsruhe Institute of Technology, Karlsruhe, Germany; 6 German Center for Neurodegenerative Diseases (DZNE), Witten, Germany; 7 German Center for Neurodegenerative Diseases (DZNE) Site Rostock/Greifswald, Rostock, Germany; 8 Department of Psychiatry and Psychotherapy, Medical Faculty, University of Cologne, Cologne, Germany; 9 WBS Training, Schwerin, Germany; 10 Department of Psychiatry and Psychotherapy, University Medical Center Goettingen, Goettingen, Germany; 11 German Center for Neurodegenerative Diseases (DZNE), Goettingen, Germany; 12 PP.Rt, Clinical Center of Psychiatry and Psychosomatics, Reutlingen, Germany; 13 Institute of Social Medicine and Health Systems Research, Faculty of Medicine, Otto-Von-Guericke University, Magdeburg, Germany; 14 Rostock University Medical Center, Rostock, Germany; 15 IKK Gesund Plus, Magdeburg, Germany; 16 Department of Psychology, University of Konstanz, Constance, Germany; 17 McMaster University, Hamilton, Canada

**Keywords:** Dementia, Dementia care management, Collaborative care, Informal caregivers, Caregiver burden, Mobile app, MHealth app, Randomized controlled trial

## Abstract

**Background:**

Informal caregivers provide a large part of the care required by people living with dementia. With disease progression, the burden on caregivers typically increases. Dyadic interventions, addressing both the caregiver and the person living with dementia simultaneously, can help to stabilize home care settings. This study examines the effectiveness and cost-effectiveness of an app-assisted dementia care intervention.

**Methods:**

*Living@home* is a two-arm, randomized, controlled, multi-center interventional study designed to evaluate the effectiveness of a dyadic, app-based intervention on home care stability as well as the cost-effectiveness of this approach. The inclusion criteria are a formal diagnosis of dementia and living in their own home for the person with dementia, as well as access to a smartphone and internet for the informal caregiver. A total of 554 dyads will be recruited in five participating memory clinics and randomized (1:1 ratio, using block randomization) into either the intervention or the control group. The intervention group will receive personalized dementia care management for a period of 12 months. A mobile health app connecting caregivers with a Family Care Specialist offers informal caregivers access to memory clinics and Care Specialists at any time, monitoring of their health situation through real-time data collection, overview of care-related tasks, information and alerts tailored to the reported individual caregiver burden. The control group receives care-as-usual. Both groups will be assessed at baseline and after 12 months. The primary outcome is the caregiver’s perseverance time (Perseverance Time Scale), and crucial secondary supportive outcomes are neuropsychiatric symptoms in the person living with dementia (Neuropsychiatric Inventory), and the caregiver’s perceived burden (Zarit Burden Interview). Further secondary outcomes include the caregiver’s resilience and self-efficacy, the dyads’ physiological stress level (as measured by hair cortisol concentrations), health-related quality of life, caregiver depression and anxiety, dyads’ unmet needs, and cost-effectiveness. Intervention effects will be estimated by using multivariate regression analyses.

**Discussion:**

Evidence on the effectiveness and cost-effectiveness of the intervention created from this study can help to inform decision makers and support the transition of the intervention to standard care.

**Trial registration:**

ClinicalTrials.gov NCT07251088, Registered 25.11.2025

## Introduction

### Background and rationale {6a}

In Germany, approximately 84% of the five million individuals in need of care are supported at home by informal caregivers, typically family members. Among these, only 21% receive professional assistance from care or support services, underscoring the substantial burden on informal caregivers [1]. People living with dementia (PlwD) represent a large share of these care recipients, with an estimated 1.8 million individuals affected. On average, they require 36 h per week of informal care—significantly more than individuals with severe diagnoses, such as cancer (16 h/week) or stroke (24 h/week) [2, 3].

Most PlwD and their caregivers (hence referred to as dyads) express a strong desire to remain in their home environment and continue home-based care for as long as possible [4]. Due to the progressive nature of dementia—including increasing cognitive and physical impairments as well as neuropsychiatric symptoms—informal caregivers are continuously challenged to respond to changes in the care situation and to adapt home-based care arrangements to meet evolving needs [5]. Although many caregivers find meaning in their role, the intensive support and care of people living with dementia often lead to both physical and psychological strain on caregivers. This is often associated with depression, anxiety, lower subjective well-being, reduced self-efficacy, and limited social participation [3, 6–9]. Consequently, the sustainability of home care is frequently unstable [10]. Therefore, forty percent of caregivers of community-dwelling PlwD report that they are unable to maintain home care for more than 1 year [11], often resulting in institutionalizations.

Although support services for caregivers have been continuously expanded in recent years, utilization rates remain low [12], often due to a lack of awareness or organizational barriers [13]. Moreover, caregiver workload itself hampers the use of such services. Without adequate support, informal caregiving is increasingly replaced by professional long-term care, placing additional pressure on the scarce personnel and financial resources of the healthcare system [12] and frequently eliciting feelings of guilt in caregivers [13].

To date, randomized controlled intervention trials have only partially demonstrated significant effects on caregiver burden [6]. A meta-analysis revealed that individualized and structured multicomponent interventions, addressing various domains simultaneously, were most effective in reducing caregiver burden [14]. Evidence for the cost-effectiveness of such interventions exists but is limited due to small sample sizes [15]. A model-based analysis suggests that dyadic interventions targeting both PlwD and caregivers may prolong the ability to sustain home care and could potentially be cost-effective [16]. However, findings are mixed, as a meta-analysis of randomized controlled trials on dyadic interventions reported 13 studies with positive outcomes and no effect in 9 others [17]. One reason for the observed inconsistencies may be that the effectiveness of interventions appears to depend on their duration and intensity. Possibly, caregiver needs at times of increasing burden were not always adequately or promptly enough addressed [17]. Digitally supported approaches, such as remote, app-based interventions, could help overcome some of these limitations by providing flexible, immediate support whenever it is most urgently needed. However, research on such digital interventions remains sparse, and it remains unclear to what extent these tools can truly stabilize home care situations.

### Objectives {7}

The primary objectives of the *living@home* study are (1) to assess whether a dyadic care management approach carried out by specialized nurses (Family Care Specialists) combined with the use of a mobile health app providing direct access to a support system (Care Specialists and memory clinics) can stabilize the home care situation, (2) to reduce caregiver burden, and (3) to alleviate neuropsychiatric symptoms in PlwD compared to care as usual.

### Trial design {8}

The *living@home* study is a multi-center, randomized, controlled intervention study with two parallel arms: an intervention arm and a control arm (care as usual). Dyads in the intervention group will receive an individualized dementia care management for a period of 12 months, aiming to identify and effectively address the unmet needs of PlwD and their caregivers. The intervention will be supported by a mobile health app used by both caregivers and Care Specialists, enabling caregivers to access care managers and memory clinics at any time when needs or burdens are faced. Therefore, the app will be used to monitor the caregiver’s health situation and burden through regular real-time data collection, aggregation, and transmission to the care manager for timely interventions or re-interventions tailored to the individual caregiver’s burden. The core items and their respective cut-offs are summarized in Table 1.

**Table 1:** App-based monitoring and stratifaction

Item	Source/reference	Cut-off/threshold	Contact with family caregiver	Reassessment frequency
High caregiver distress	PTS and ZBI-12 and ZBI-1	PTS < 6 months or ZBI-12 ≥ 20 or ZBI-1 “extremelx”	Weekly intervention contact	Monthly
Moderate caregiver distress		PTS < 6 and > 24 months or ZBI-12 < 20 or ZBI-1 “quite a bit”	Monthly intervention contact	Monthly
Low caregiver distress		PTS < 24 months or ZBI-12 < 10 or ZBI-1 “not at all/little/moderately”	Quarterly intervention contact	Quarterly

**Fig. 1:**
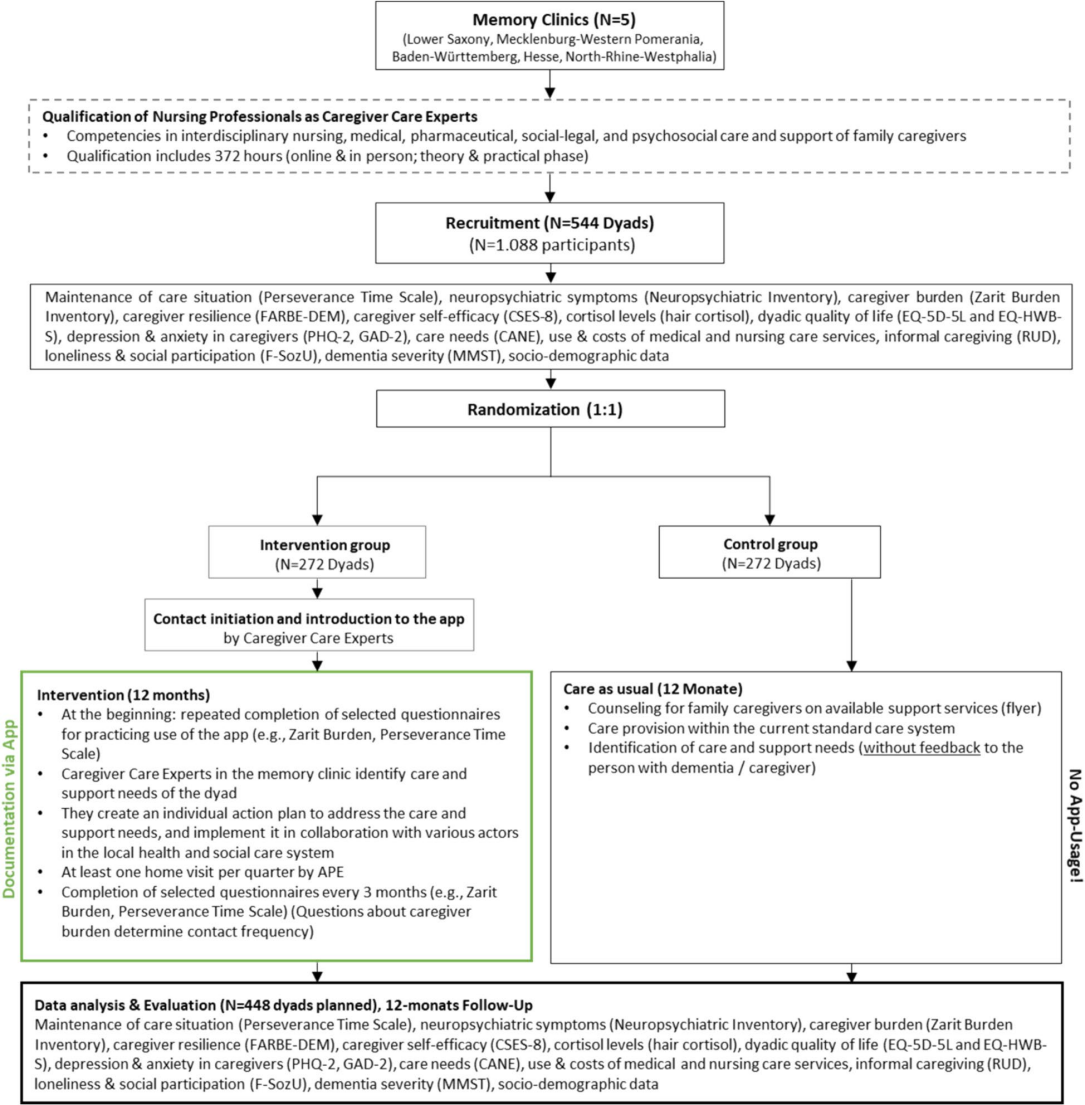
Schematic overview of trial design

Thus, the intervention frequency and intensity are individualized to the dyads’ needs and the reported burden. Dyads in the control group will receive care as usual. Both groups will be assessed at baseline and after 12 months. The study flowchart is illustrated in Fig. 1.

## Methods: participants, interventions, and outcomes

### Study setting {9}

The study is set within the patients’ homes and the context of specialized outpatient healthcare provision (memory clinics) in Germany.

### Eligibility criteria {10}

Eligible participants are dyads consisting of a PlwD and an informal caregiver. The PlwD must have a formal diagnosis of dementia and reside in his/her own home. The informal caregiver must be the primary caregiver of the PlwD with access to a mobile device with online capabilities. The dyad must reside within the catchment regions of the participating memory clinics to facilitate home visits.

### Who will take informed consent? {26a}

Participating dyads will receive all relevant information on the study’s objectives, procedures and risks before registration. Both the PlwD and the informal caregiver need to give written informed consent prior to the study. The written study information and the consent form are adapted for the two participating groups; for example, for the PlwD, the forms are reformulated in a simplified format. If the PlwD is unable to consent, their legal representative will be asked to provide written informed consent on their behalf. Physicians in the recruiting memory clinics will be responsible for ensuring the PlwD’s ability to provide informed consent. The original declarations will be kept safely at the respective study center. Participants (and caregivers) will receive a copy. Study participants can withdraw their consent at any time without negative consequences for them. In case of revocation, all personal data of the participant will be anonymized or deleted, if necessary (at DZNE).

### Additional consent provisions for collection and use of participant data and biological specimens {26b}

Participants may choose to consent or decline to provide a hair sample for cortisol-level analysis.

## Interventions

### Explanation for the choice of comparators {6b}

Dyads in the control group will receive care as usual, reflecting the current standard of care in German memory clinics and subsequent outpatient treatment and care.

### Intervention description {11a}

#### Intervention design

The intervention consists of a structured, individualized, dyadic, collaborative approach, identifying and addressing the needs of both the PlwD and the informal caregiver. Family Care Specialists (qualified nurses) identify the care and support needs of the dyad using a tablet-based intervention management system (IMS).

In general, the intervention consists of four key components:

i. Initial needs assessment at the memory clinic, followed by a home visit with a comprehensive initial resource and need assessment of the dyads in their current home-living situation. In these assessments, comprehensive information regarding the medical, psychological, pharmaceutical, social and nursing situation is collected to identify the individual resources and unmet needs of both the caregiver and the PlwD. The information collected informs the subsequent selection of intervention tasks, which are then tailored by the Family Care Specialist to the dyad’s context and preferences.
ii. Generation of an individual intervention task list based on the identified unmet medical, psychosocial, and care-related needs. While the system does not explicitly assess personal life history, the Family Care Specialist evaluates the suitability of the suggested tasks for the dyad and adapts them as necessary to match the dyad’s capabilities, preferences, and context.
iii. Collaborative completion of the task list by the Family Care Specialist in close cooperation with the treating general practitioner of the caregiver and the PlwD and other healthcare and social services providers involved in the care for either the caregiver, or the PlwD, or both during the 12-month intervention period. Tasks are reviewed and adjusted collaboratively with the dyad to ensure they are feasible and appropriate. The Family Care Specialist may also add tasks not predefined in the IMS if these better align with the dyad’s needs and preferences.
iv. Access to a mobile health app for caregivers for digital support, caregiver burden monitoring, and connection to the Care Specialist and the memory clinic at any time, enabling timely response as well as identification of emerging unmet needs and additional intervention tasks.

The content, the frequency and intensity of the intervention tasks are adjusted according to the caregiver’s burden and the stability of the home care situation, meaning that the higher the burden or the lower the home care stability, the more frequent and intense the intervention will be. Dyads with a low caregiver burden (ZBI-12 < 10 or ZBI-1 “not at all/little/moderately”) and a stable home care arrangement (Perseverance Time Scale > 24 months) are considered as low strain and will be contacted by the Family Care Specialist on a quarterly basis to check on their needs. Dyads with a moderate to high caregiver burden (ZBI-12 10–19 or ZBI-1 “quite a bit”, and ZBI-12 ≥ 20 or ZBI-1 “extremely”) or a moderate to low care arrangement stability (Perseverance Time Scale < 24 months and > 6 months, and < 6 months) are considered as medium to high strain resulting in a monthly or weekly contact frequency, respectively.

Besides these regular contacts (executed via a visit at the home of the PlwD or the caregiver or a phone call or a video call via the mobile health app) the caregivers can reach out to the Family Care Specialists anytime to seek assistance or advice. 

#### Intervention management system

Based on the assessed needs, the Family Care Specialists develop an individualized action plan and implement it in collaboration with various healthcare and social services providers operating within the PlwD’s home environment.

The implementation of the intervention is supported by a computerized IMS, a rule-based expert decision support tool to help Care Specialists in identifying PlwD’s and caregiver’s unmet needs and selecting suitable interventions. Via predefined algorithms and based on PlwD’s and caregiver’s responses to predefined questions from the needs assessment, the system will automatically identify unmet needs and suggest appropriate interventions to address these needs, all summarized in a generated intervention task list. It also supports the intervention monitoring. The IMS draws upon components from prior trials, including the DelpHi-MV [18], Independent [19] and GAIN trial [20]. For *living@home*, the IMS system was further developed by integrating dyad-specific modules and revising all intervention recommendations through multiple expert rounds involving healthcare and nursing scientists, clinicians, and Care Specialists.

#### Mobile health app

An additional key component of the intervention is the mobile health application, which is available to all participating caregivers. The app is used to execute repeated short assessments of the stability of the care situation and the caregiver burden, based on which the dyads are stratified into three intervention intensity levels. This ensures that dyads with a higher caregiver burden or less stable care arrangement receive more intensive support from the caregivers. Also, the level of strain determines the recurrence of the app assessments: dyads in the low-strain group are re-assessed via the app quarterly, while dyads in the moderate and high-strain groups are re-assessed monthly. Besides the tool for repeated assessment, the mobile health app contains several functionalities, such as a chat feature for messaging between the caregiver and the Family Care Specialist, a caregiver diary to capture health and life events, a digital library containing educational materials provided as videos, information text with links that help to address existing unmet needs, and a regional location guide for dementia-related support services.

### Criteria for discontinuing or modifying allocated interventions {11b}

Since the intervention is individualized, its implementation may differ between dyads. However, modifications will be made in specific circumstances: if the caregiver dies, the intervention may be adapted to involve a new caregiver, or the PlwD may continue participation independently. If the PlwD dies, the study will be discontinued for the caregiver. The same procedure applies if either member of the dyad withdraws consent.

### Strategies to improve adherence to interventions {11c}

The intervention is individualized to the dyad’s needs and delivered collaboratively. Continuous access to the intervention and the mobile health app allows caregivers to seek timely support, advice and assistance, promoting engagement and sustained participation. The app’s interactive features—such as regular assessments and direct communication—are designed to foster commitment to the intervention and reduce attrition.

### Relevant concomitant care permitted or prohibited during the trial {11d}

The intervention is subsidiary to usual care. Therefore, all forms of routine medical treatment and psychosocial care available within the German healthcare system will be permitted during the trial. No specific treatments are prohibited.

### Provisions for post-trial care {30}

No specific post-trial care is planned, given the non-invasive and low-risk nature of the intervention. The total study period, during which interventions will be conducted for dyads, is 30 months. During this time, dyads entering the study early may continue to use the app and remain in contact with the informal-caregiver expert after the official 12-month intervention period.

### Outcomes {12}

Primary and secondary outcomes will be assessed at baseline and 12 months after baseline by qualified study nurses not knowing the group assignment via face-to-face interviews and by caregiver self-assessments at the participating memory clinics, allowing for blinded assessment. Furthermore, baseline assessments will be conducted before randomizing dyads to the intervention or control group, also supporting blinding.

#### Primary outcome

The primary dyadic outcome is the caregiver’s self-assessment of the stability of the home care arrangement (Perseverance Time Scale) [11, 21]. Crucial secondary supportive outcomes are the presence, type and severity of neuropsychiatric symptoms of the PlwD (Neuropsychiatric Inventory) [22] and the caregiver burden (Zarit Burden Interview) [23, 24]:

i. For the Perseverance Time Scale, informal caregivers are asked (self-assessment) to estimate how long they believe they can continue to provide informal care and, thus, their current care situation. Response options are less than 1 week, less than 1 month, less than 6 months, less than 1 year, less than 2 years, and 2 years or more.
ii. The caregivers will also report on the Neuropsychiatric Inventory via face-to-face interviews with a qualified study nurse, providing details about the presence and severity of neuropsychiatric symptoms, including depression, aggression, hallucinations and other behavioral symptoms of the PlwD.
iii. The Zarit Burden Interview (12-item version) and ZBI-1 will be used to measure caregiver burden via a self-assessment, measuring physical and emotional strain, social restrictions, financial burden, and perceived effects on caregivers’ health.

The primary outcome is the stability of the home-based care arrangement, measured by the Perseverance Time Scale which operationalizes the caregivers’ subjective self-estimation of their ability to sustain the current home-based care arrangement. While caregivers’ predictions about future care capacities may be uncertain due to the chronic and progressive nature of the disease, the Perseverance Time Scale is short, easily applicable and well-validated for use in this population. As demonstrated by previous studies with informal caregivers of PlwD, the Perseverance Time Scale showed significant correlations with other burden-related measures, such as the Caregiver Strain Index, the Self-related Burden Scale or the CarerQol-VAS [25]. Changes in caregiver-reported outcomes will be contextualized with concurrent events, such as functional decline of the PlwD, hospitalizations, falls, or other health-related incidents, allowing for a more comprehensive understanding of care dynamics over time.

A crucial secondary supportive outcome is the presence of neuropsychiatric symptoms in PlwD, assessed with the Neuropsychiatric Inventory questionnaire, completed by the informal caregiver via face-to-face interviews with the study nurse. Neuropsychiatric symptoms are well-documented contributors to caregiver burden [26], underlining the dyadic nature of selected primary and crucial secondary supportive outcomes. Caregivers of PlwD exhibiting a higher number of neuropsychiatric symptoms are known to be at elevated risk for emotional and physical strain [27–29] with irritability, agitation/aggression, sleep disturbances, anxiety, apathy, and delusion having the most significant effect on caregiver burden [30]. In the present study, the NPI serves as a standardized outcome measure to quantify behavioral symptom frequency, severity, and associated caregiver distress. Within the intervention, however, behavioral changes are interpreted contextually in relation to the dyad’s care environment. This perspective enables behavioral expressions to inform care planning in a needs-oriented and person-centered manner.

Another crucial secondary supportive outcome is the caregiver burden itself, measured via the Zarit Burden Interview (12-item version), which will be self-completed by caregivers. This tool is among the most commonly applied instruments in informal dementia caregiver research, enabling comparability across studies. While the most commonly used form of the Zarit Burden Interview is a 22-item version, the shortened 12-item version and the 1-item version have demonstrated comparable psychometric properties [23, 24], offering a time-efficient alternative that reduces respondent burden.

#### Qualitative data

In addition to the quantitative analyses, we will conduct guided interviews with experts who are involved in the care process (e.g., physicians in specialized care) to assess facilitators, barriers, acceptance, usability and feasibility of the intervention. In addition, the usability of the Mobile Health App and the Intervention Management System will be evaluated. Interviews will be conducted with *n* = 8 participating dyads, *n* = 8 Family Care Specialists, and *n* = 3 physicians from participating memory clinics. The qualitative data will be evaluated using content analyses by Mayring [31].

#### Further secondary outcomes

A secondary outcome of the study is the caregiver’s resilience, measured by the Resilience and Strain Questionnaire in Caregivers of People with Dementia (ResQ-Care-Dem) questionnaire, which consists of four scales with five items each. Two scales (“My inner attitude” and “My sources of energy”) assess resilience [32, 33]. The other two (“Difficulties in managing the person with dementia” and “General burdens of my living situation”) assess perceived burden. The instrument creates different profiles based on the ratio of burden and resilience factors.

To measure objective stress, hair samples will be analyzed by the University of Konstanz in collaboration with the University of Vienna to assess hair cortisol concentration in caregivers and PlwD, providing a physiological marker of chronic stress [34]. For this analysis, the study nurse will collect two strands of hair from each participant’s back of the head (posterior vertex) at baseline and 12 months later. The strands together should have a diameter of half a pencil, a length of at least 3 cm, and they should be cut as close to the scalp as possible. In addition, information regarding the participant’s hair (e.g., natural color, hair treatments, washing frequency) will be collected via questionnaire. If participants do not consent to the capturing of a hair sample or if their hair is shorter than 3 cm, no samples will be taken.

Another secondary outcome is the caregiver’s self-efficacy measured with the CSES-8 [35]. Self-efficacy means the strength of the caregiver’s belief in being able to complete a specific task. It is a modifiable trait, making it a valuable secondary outcome. The instrument creates a mean score of eight items, with higher scores indicating greater confidence in one’s own caregiving abilities.

A high caregiver burden, less resilience factors and low self-efficacy can lead to depression or anxiety symptoms [36]. Thus, these symptoms will be screened using short versions of the Patient Health Questionnaire (PHQ-2) and the Generalized Anxiety Disorder (GAD-2) tool. Both instruments yield sum scores between 0–6, with ≥ 3 indicating the likely presence of the respective disorder.

As the intervention supports dyads in reducing unmet needs, a secondary outcome is the measurement of unmet needs among PlwD and their caregivers using the Camberwell Assessment of Need for the Elderly (CANE). It consists of 27 categories (including two categories for the caregiver), which assess participants’ needs in various domains. Initially, each of the categories is described (e.g., living situation, household, nutrition, personal hygiene) followed by a question whether the participant has an unmet need in this area (Yes, No) [37]. The CANE is available in two versions: participant and caregiver. If the participant is not able to answer the questions, the caregiver will be asked about the open needs of their relative or caregiver. CANE has been determined suitable for scientific use in dementia and demonstrates appropriate criterion validity [38]. It was easily used by a wide range of professionals without formal training.

#### Process evaluation

The intervention will also be evaluated in terms of acceptance and satisfaction from the perspectives of PlwD, caregivers, and care managers through qualitative interviews within a process evaluation.

#### Claim data analysis

Secondary data, delivered by two participating statutory health insurers, will be used to compare healthcare utilization between dyads being treated at a memory clinic and those receiving care through other institutions (other than memory clinics). The outcomes of this separated analysis of the secondary data—without individual data linkage to primary data—will be the progression of care level for the PlwD and the frequency of mental health problems (ICD-10 diagnoses) for the relatives.

#### Health economic evaluation

For the health economic evaluation, we will measure the dyads’ health-related quality of life by applying the EQ-5D-5L [39] and the EQ-HWB-S [40] as patient and caregiver self-ratings within interviews. The EQ-5D-5L is a widely used, standardized generic instrument that measures the respondent’s health-related quality of life in five dimensions. Responses can be summarized in a health profile that is associated with a health utility index, ranging from zero (death) to one (full health). In combination with the patient’s and caregiver’s life expectancy, the index can be used to calculate quality-adjusted life years (QALYs) for both the patient and the caregiver. The EQ-HWB-9 (EQ Health and Wellbeing short version) is a new generic instrument to measure health and well-being for social care recipients and caregivers and captures a range of health and broader quality of life aspects in seven dimensions.

Healthcare resource use, which is also required for the health economic evaluation, is captured using the Questionnaire for Health-Related Resource Use in an Elderly Population (FIMA) [41] and the Resource Utilization in Dementia Questionnaire (RUD) [42]. These instruments assess the use of medical, formal, and informal care resources, enabling cost calculation from both a payer’s and a societal perspective.

### Participant timeline {13}

Participant recruitment will take place from January 2026 to July 2027. After recruitment and baseline assessment, dyads will be randomized. Dyads of the control group will receive the intervention for a duration of 12 months. The frequency of the intervention (meaning interactions with the Family Care Specialist) will be determined individually based on the stratification into low-, medium-, or high-strain group. Twelve months after baseline, the dyads will receive a follow-up assessment. The last intervention will thus be carried out in July 2028.

### Sample size {14}

Sample size was exclusively calculated based on a dichotomized threshold (≥ 12 months) of the PTS. Based on data from previous studies, 60% of informal caregivers of community-dwelling PlwD initially expressed their confidence in maintaining the informal care provision and the current care arrangements for at least 12 months. However, 10.5% of patients died and only 23% of the initial sample maintained this view one year later [21]. We, therefore, hypothesize that the intervention will increase this proportion to 35%, corresponding to an effect size of 0.25. Assuming a 1:1 randomization, statistical power of 80%, and a significance level of *α* = 0.05, a sample size of 224 dyads per group is required to detect a statistically significant effect based on a chi^2^ test. Considering an additional drop-out rate of 17.5%, summing up to 28% including death (in line with previously conducted study [43]), the target sample size increases to 272 dyads per group, resulting in a total of 544 dyads (1088 participants: 544 PlwD and 544 caregivers).

### Recruitment {15}

Recruitment will be conducted in five participating memory clinics over 18 months. To reach the goal of 544 dyads in total (108 per study center), each clinic must recruit six dyads per month. To mitigate the risk of under-recruitment, several strategies may be employed, including allowing over-recruitment at individual sites to compensate for under-recruitment at others, engaging additional memory clinics proximate to the current study centers into recruitment activities, sending study invitations to potentially eligible participants via collaborating health insurance providers, maintaining a “waiting list” of interested dyads who contact the clinic up to 6 months before the recruiting phase, and informing and involving general practitioners in each region to support referrals. Participants who provide written informed consent to participate will subsequently be included in the study.

## Assignment of interventions: allocation

### Sequence generation {16a}

The randomization will be conducted by the University Medicine Greifswald, Institute for Community Medicine using RandList software (Version 2, Datinf, Germany). Eligible dyads will be randomly assigned in a 1:1 ratio to the intervention or control group using computer-generated block randomization at the level of the memory clinics to ensure the parallelism of sampling, even in the case of under-recruitment. The clinics will receive the randomization lists in blocks of six.

### Concealment mechanism {16b}

The allocation sequence will be performed by the evaluation team using RandList software. The randomization list will be accessible only to designated personnel within the project’s evaluation (internal) team of the Institute of Community Medicine (University Medicine Greifswald) and will be revealed after the baseline assessment is completed. All other study staff and investigators will have no access to the randomization list.

### Implementation {16c}

The allocation sequence will be performed by the Institute of Community Medicine (ICM) using RandList software.

## Assignment of interventions: blinding

### Who will be blinded {17a}

Study nurses capturing the outcomes will be blinded. However, due to the nature of the intervention, blinding is not feasible during the intervention period for Family Care Specialists and other staff of the memory clinic. Randomization will occur only after recruitment and baseline assessment are finalized. By maintaining concealment until this point, we mitigate potential selection bias and ensure that group assignment does not influence the initial evaluation of the dyads. Both caregiving experts and participating dyads will become aware of their group assignment once the intervention begins. As a result, the study is conducted with partial blinding, limited to the baseline assessment.

### Procedure for unblinding if needed {17b}

Not applicable.

#### Data collection and management

### Plans for assessment and collection of outcomes {18a}

After study inclusion, all participants will receive a baseline assessment using structured face-to-face interviews conducted by qualified study nurses, as well as caregiver self-assessments at the respective memory clinic. To comprehensively assess both individual and interactive aspects of caregiving, primary and crucial secondary supportive outcomes reflect the perspectives of both the PlwD and the informal caregiver. Follow-up assessment will be carried out 12 months after the baseline assessment in both study groups.

All study personnel involved in data collection have received intensive online training not only in data collection procedures, but also in communication with PlwD and their caregivers to ensure that the data collection process is not overly burdensome for participants and enable them to detect signs of distress and respond appropriately.

### Plans to promote participant retention and complete follow-up {18b}

Dyads in the intervention group will maintain frequent contact with the Family Care Specialists through home visits, phone calls, and messages via the app. The app will periodically prompt caregivers to complete brief questionnaires, and the caregiver’s reply will possibly trigger contact between the Care Specialist and the caregiver. Continuous access to the intervention and the mobile health app allows caregivers to seek timely support, advice and assistance, promoting engagement and sustained participation. The app’s interactive features—such as regular assessments and direct communication—are designed to foster commitment to the intervention and reduce attrition.

Dyads in the control group will receive a financial incentive (50 € per participant) for each completed baseline and follow-up interview. 

### Data management {19}

To ensure high data quality and completeness of data sets, multiple measures will be implemented during the study: before data entry, study nurses and Family Care Specialists receive comprehensive training on data handling and entry, using case examples and a test environment in the IMS software. At project initiation, a structured data monitoring system based on eCRFs within an IT-supported documentation platform will be established. This system employs intensive monitoring and key performance indicators to continuously ensure high data quality. Data management by a medical documentalist includes automated tasks, reminders for pending data collection, and systematic checks for completeness, quality, and plausibility. Any irregularities or missing data trigger feedback to study nurses and Family Care Specialists referring to participant IDs. This process ensures ongoing optimization of data collection. Additionally, designated contacts for data entry issues are communicated clearly to all study personnel at the start of the study. A regular coordination meeting (Jour Fixe) between the Family Care Specialists and the evaluation team facilitates early identification and resolution of data entry challenges as well as periodic retraining throughout the study.

### Confidentiality {27}

All data will be stored on secure servers, with personal identifiers kept separate from participants’ health information. Data storage complies with current standards for data security and privacy in accordance with the General Data Protection Regulation (GDPR). Only Family Care Specialists, study nurses, experts and other staff involved in the study (e.g., clinicians) will have access to personal data during baseline and follow-up assessments. An administrator will take care of the pseudonymization. All further study personnel will only get access to pseudonymized or aggregated data.

Paper documents (e.g., informed consent forms) will be securely stored and accessible only to authorized personnel. Access to the study software is protected by individual user passwords and a detailed rights and roles system, which emphasizes data minimization and security. Family Care Specialists and study nurses will only be able to view participant information relevant to their assigned memory clinic. All tablet PCs used for data collection are also secured with individual passwords.

### Plans for collection, laboratory evaluation and storage of biological specimens for genetic or molecular analysis in this trial/future use {33}

Not applicable.

## Statistical methods

### Statistical methods for primary and secondary outcomes {20a}

All data analyses will be conducted using pseudonymized datasets. Initially, all outcome variables will be subject to descriptive statistical analyses. At baseline, the intervention and control groups will be compared regarding significant differences, serving as a randomization check. If notable differences are identified, all subsequent analyses will be adjusted for the respective factors. After completing the assessment, we will conduct a drop-out analysis using multivariate logistic regression to evaluate any systematic differences between included and excluded patients.

For the primary outcome, an ordinal logistic regression (proportional odds model) will be used to analyze the full six-category ordinal scale PTS. The intervention effect on the secondary outcomes will be evaluated using multivariate linear and logistic regression models, adjusted for age, sex, and living situation (alone vs. together with the caregiver), and including random effects for the respective memory clinic to account for clustering. The different study sites will be treated as clusters in these analyses. The analyses will be done on intention-to-treat (base case analysis) and per-protocol analyses (sensitivity analysis).

For the health-economic evaluation, the incremental cost-effectiveness ratio (ICER) will be calculated by comparing the additional cost per QALY gained through the intervention. Nonparametric bootstrapping resampling will be used to account for sample uncertainty in the ICER estimates. Various willingness-to-pay thresholds will be applied to determine probabilities of cost-effectiveness.

#### Qualitative analysis

Interviews will be audio-recorded and transcribed using MAXQDA software (version 12.0 or later, VERBI, Berlin, Germany). Using a qualitative content analysis approach by Mayring, the transcribed data will be analyzed [31]. The evaluation process will be conducted in accordance with the Medical Research Council’s 2006 guidelines [44].

### Interim analyses {21b}

Not applicable.

### Methods for additional analyses (e.g., subgroup analyses) {20b}

Quantitative outcomes within subgroups will be analyzed using multivariate linear and logistic regression models. If differences between the intervention and control groups are observed at baseline, adjustments will be made for relevant covariates such as age, gender, and dementia severity. In addition to intention-to-treat analyses, per-protocol analyses will be conducted with participants who actually received the intervention. Further analyses will focus on specific subgroups of the study population, for example based on dementia severity or variations in home care settings.

### Methods in analysis to handle protocol non-adherence and any statistical methods to handle missing data {20c}

Missing data will be reported and described in all descriptive analyses. For multivariate analyses, missing data may be imputed using multiple imputation via chained equation, stratified by the respective group, when the missing data are at random.

### Plans to give access to the full protocol, participant-level data and statistical code {31c}

Participant-level data, statistical codes, and the full protocol may be made available upon reasonable request. Access will be managed following institutional policies and ethical approvals.

## Oversight and monitoring

### Composition of the coordinating centre and trial steering committee {5d}

A consortium of several partners is forming the trial management group. Five memory clinics (Rostock, Göttingen, Cologne, Darmstadt, Reutlingen) are responsible for recruitment, implementation of the intervention, and data collection. The Karlsruhe Institute of Technology is responsible for designing, implementing and maintaining the mobile health app. The Institute for Community Medicine at the University Medicine Greifswald is responsible for the evaluation of the study (both effect and process evaluation). The University of Konstanz is responsible for the hair cortisol analyses, while the analyses of the secondary data are done by the Institute of Social Medicine and Health Systems Research, University of Magdeburg. Furthermore, two statutory health insurers are part of the group and take care of the development of the legal framework for the care model as well as the corresponding contracts. Also, they provide the required claims data for analyses. Regular meetings are held between all partners to coordinate all required processes.

### Composition of the data monitoring committee, its role, and reporting structure {21a}

The objective evaluation of the study is ensured by the Institute for Community Medicine (University Medicine Greifswald), which has an external and independent role to the consortium of the trial. All results will be submitted for peer-reviewed publication.

### Adverse event reporting and harms {22}

Although adverse effects are not expected from the dementia care intervention, any adverse events or possible unintended effects will be recorded, evaluated and communicated to all relevant stakeholders. Previous studies have shown that similar dementia care management interventions are associated with minimal to no harm and can be implemented safely.

### Frequency and plans for auditing trial conduct {23}

Not applicable.

### Plans for communicating important protocol amendments to relevant parties (e.g., trial participants, ethical committees) {25}

Any protocol modifications, as well as relevant process changes, will be communicated to all involved ethics committees and noted in the study protocol. All changes will be noted in the study registration as well.

### Dissemination plans {31a}

All relevant study results will be submitted for publication in peer-reviewed scientific journals and presented at scientific conferences and meetings. All study partners and participating health service providers will also receive reports on the published results. Reports of all results will also be shared with the funding agency (G-BA).

## Discussion

The rising number of PlwD due to demographic change, as well as the absence of available and effective treatment, poses a challenge for health care systems worldwide in the coming decades. Due to the limited availability of personnel resources and financial constraints, informal caregivers continue to play an important role in sustaining adequate care services for PlwD. With growing dependence and need for care due to the progressing nature of dementia, the burden on the caregivers increases and may destabilize the home care arrangement. The consequence is often an institutionalization of the PlwD. The *living@home* intervention aims to provide support to both the PlwD and the informal caregiver to enable them to maintain the informal care provision and living at home running as long as possible. This aim is to be achieved by implementing a Dementia Care model, which provides dyadic, subsidiary care and support services. The intensity of the intervention and support measures will be individually adapted to the burden of the dyad, helping to allocate financial and personnel resources efficiently.

The project’s aim is to generate robust evidence on the effectiveness and cost-effectiveness of the intervention in stabilizing home care arrangements and reducing the burden on informal caregivers. Positive results will support the transition of the intervention to standard care. Since the trial will be conducted within a real-life setting in memory clinics, the results will be of high validity and likely generalizable to settings in all memory clinics in Germany.

### Limitations

A notable limitation of this study is the lack of participant and caregiver blinding, which is often unavoidable in complex psychosocial real-life interventions. This open-label design introduces a risk of performance and detection bias, as caregivers in the intervention group were aware of the additional support provided by the app and Family Care Specialists. Such awareness may result in a positive reporting bias (Hawthorne effect) [45], where participants might overestimate their perceived resilience (PTS) or underestimate their burden (ZBI) due to social desirability or high expectations. We estimate the potential magnitude of this bias to be moderate.

To mitigate this risk and ensure the robustness of our findings, we employed a triangulation strategy. By integrating objective secondary outcomes—specifically physiological hair cortisol levels as a marker of chronic stress and health insurance claims data to track the progression of care levels—we provide an empirical counterweight to the self-reported measures. The alignment of these objective parameters with subjective assessments allows for a more reliable interpretation of the intervention’s true clinical impact, reducing the likelihood that the observed effects are merely a result of reporting artifacts. 

## Trial status

The current protocol is version 2.0, dated 20.02.2026. Recruitment started in January 2026 and is expected to continue until July 2027.

## Data Availability

Access to the final dataset with anonymized data will be available to all members of the trial consortium.

## Abbreviations

CANE: Camberwell Assessment of Need for the Elderly
DZNE: Deutsches Zentrum für neurodegenerative Erkrankungen/German Center for neurodegenerative diseases
ICER: Incremental cost-effectiveness ratio
IMS: Intervention management system
PlwD: People living with dementia
QALY: Quality-adjusted life year
